# Full versus partial thickness flap to determine differentiation and over keratinization of non‐keratinized mucosa. A 3‐year split mouth randomized pilot study

**DOI:** 10.1002/cre2.468

**Published:** 2021-07-10

**Authors:** Algirdas Puisys, Viktorija Auzbikaviciute, Egle Vindasiute‐Narbute, Saulius Zukauskas, Dainius Razukevicus, Michel M. Dard

**Affiliations:** ^1^ Vilnius Research Group VIC Clinic Vilnius Lithuania; ^2^ VIC Clinic Vilnius Lithuania; ^3^ Institute of Odontology, Faculty of Medicine Vilnius University Vilnius Lithuania; ^4^ Faculty of Dentistry Lithuania University of Health Science Kaunas Lithuania; ^5^ College of Dental Medicine Columbia University New York New York USA

**Keywords:** connective tissue graft, keratinized ginigva, soft tissue thickening, tuberosity graft

## Abstract

**Objectives:**

It was shown, that Connective Tissue Grafts (CTG) retrieved from the tuberosity tends to determine hyperplastic responses and may induce a beneficial over‐keratinization of non‐keratinized mucosa. Clinically evaluate and compare CTG from tuberosity ability to increase soft tissue thickness and the keratinization potential after recipient area is either prepared using split or full thickness flap in edentulous mandible.

**Materials and methods:**

Fourty implants were placed in 10 edentulous patients with atrophied mandible (Class IV of Misch) presenting less than 1.0 mm of keratinized tissue using a flapless approach and immediately restored with acrylic temporary bridge on multi‐unit abutments. The surgical sites were split‐mouth randomized and prepared as CTG recipients by a tunneling procedure. Twenty benefited of a partial thickness approach and 20 of a full thickness one. The CTG was placed buccally using partial thickness or full thickness flap according to the randomization schedule. The width of keratinized tissue (KT), the horizontal soft tissue thickness (STT), the marginal hard and soft tissue levels as well as the implant success parameters were collected and analyzed.

**Results:**

After a 3 year follow‐up period the increase of KT was statistically significantly (*p* < 0.001) larger in the partial thickness group from 0.6(0.6) to 5.1(0.72) mm, while full thickness group showed very little improvement from 0.5(0.51) to 1(0.57) mm (*p* < 0.001). STT was significantly increased in both groups over time: from 2.4(0.88) to 5.4(0.68) mm in full thickness group and from 2.5(0.51) to 5.8(0.41) mm in partial thickness group without any significant difference between the groups.

**Conclusion:**

The increase of soft tissue thickness by using CTG from tuberosity was found in both groups, while keratinization of non‐keratinized mucosa appeared more in the partial thickness group.

## 1 INTRODUCTION

Soft tissue grafting is widely used in implant dentistry to increase soft tissue thickness (STT) vertically (Linkevicius, Puisys, Linkeviciene, et al., 2015; Linkevicius, Puisys, Steigmann, et al., 2015; Puisys et al., 2015; Puisys et al., 2019; Puisys & Linkevicius, 2015) and horizontally (Wiesner et al., 2010) as well as the width of keratinized tissue (KT) (Cairo et al., 2019). For these purposes autogenous grafts retrieved from palate or tuberosity (Hirsch et al., 2001; Song et al., 2008; Wara‐aswapati et al., 2001) and the variety of xenogenic (Puisys et al., 2019; Thoma et al., 2020) and allogenic substitutes (Allen, 2010; Allen, 2020; Puisys et al., 2015; Puisys & Linkevicius, 2015) similar to Connective Tissue Grafts (CTG) are proposed.

The tuberosity is widely used as a donor area for soft tissue augmentation because of low morbidity, easy access and density of the graft (Burkhardt et al., 2015) which leads to the maximum of stability after implantation (Stipetić et al., 2005). It was noticed, that CTG from tuberosity tends to determine hyperplastic responses due to differences in collagen cross‐linking and the maturation of fibroblasts (Dellavia et al., 2014). This may impair aesthetic outcomes in some patients mainly related to over‐keratinization and the change of soft tissue color and texture in the aesthetic area.(Gluckman et al., 2019) Therefore, some authors recommend to use only the inner part of the palate CTG which shows a lower tendency of keratinization (Ouhayoun et al., 1988). On the other hand, this leads to difficult handling and shrinkage of the graft (Yu et al., 2013).

Partial and full thickness flaps are used for soft tissue thickening around implants (Thoma et al., 2014). It is still unclear what types of tissue will evolve after the placement of a tuberosity CTG under a full thickness or a partial thickness flap. The development of a hyperplasia and/or a keratinization has to catch up the attention of the clinician who should be prepared to properly identify these events.

The primary aim of this study was to depict and compare the appearance of a keratinization of a non‐keratinized mucosa after the CTG was placed below or above the periosteum.

The secondary aims were to evaluate and compare the soft tissue thickness and mid‐facial mucosal changes after thickening over time and between two groups.

The null hypothesis was raised that a keratinization is more evident, if CTG is placed under partial thickness flap.

## 2 MATERIALS AND METHODS

### 2.1 Study design

Ten subjects with fully atrophied mandibular alveolar ridges were enrolled consecutively from the pool of patients consulting at the Vilnius Implantology Center clinic (VIC clinic), Vilnius, Lithuania.

The study was approved by the Medical Ethical Committee of Lithuanian University of Health Science (BEC‐LSMU[R]‐71; 26 072017). All the clinical procedures were performed according to the Declaration of Helsinki for biomedical research.

Patients were eligible for the study according to the following inclusion criteria:

- males and females of at least 18 years of age.
- generally healthy patients, no medical contraindication for implant surgery.
- edentulous mandible with atrophied alveolar ridge (class IV according to Misch classification).
- no bone augmentation procedures before and during implant placement at least 5.5 mm width and 10 mm height.
- keratinized tissue width less than 1.0 mm on vestibular aspect after implant placement.
- subject had voluntarily signed the informed consent before any study related action, were willing and able to attend scheduled follow‐up visits, and agreed that pseudonymized data will be collected and analyzed.

Patients who did not fulfill the exclusion criteria were not recruited:

- Heavy smokers (more than 10 cigarettes/day).
- Systemic disease (diabetes, osteoporosis).
- Primary stability after implant placement not achieved.

### 2.2 Randomization

Randomization envelopes were used for the allocation of CTG to the 40 implants (left or right quadrant). Just after implant insertion the envelope was opened by a staff member not involved in the research protocol and analysis and the information related to CTG implant assignment was communicated to the surgeon. Depending on the draw, the CTG were placed into the gingiva tunnel under the periosteum (full thickness flap) or above the periosteum (partial thickness flap) in the same patient (split mouth design).

### 2.3 Intervention procedure

#### 2.3.1 Planning

**FIGURE 1:**
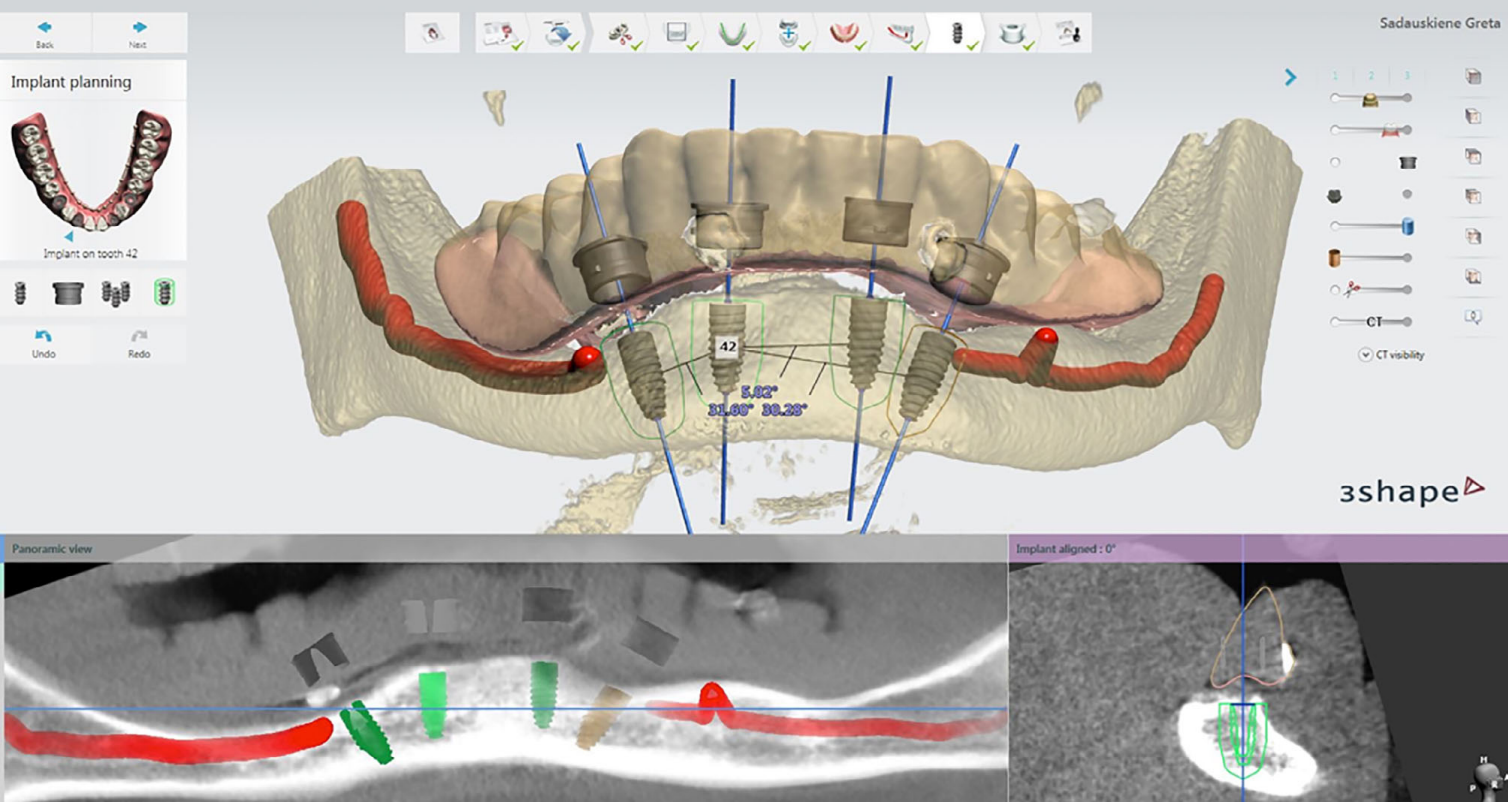
Planning of surgery, preparation of surgical guide

Diagnostic impressions using alginate material (Alligat Chroma; Heraeus Kulzer; Hanau, Germany) were made after taking intraoral and extraoral photos. Removable dentures were fabricated to restore vertical dimension and improve functional and/or aesthetical patterns. Dental Cone Beam Tomography (CBCT) scan was conducted with dentures and separately. Composite points measuring 3 mm in thickness were placed on dentures as references to match the CBCT scan and dentures by the help of an implant planning software (Implant Studio, 3 shape, Denmark). A surgical guide was designed and provided by the lab. All distal implants were tilted 30 degrees (Figure 1 Surgical guide).

#### 2.3.2 Surgery

**FIGURE 2:**
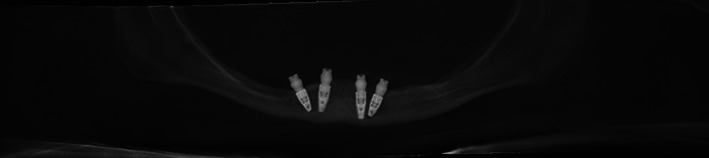
X‐ray after implant placement

**FIGURE 3:**
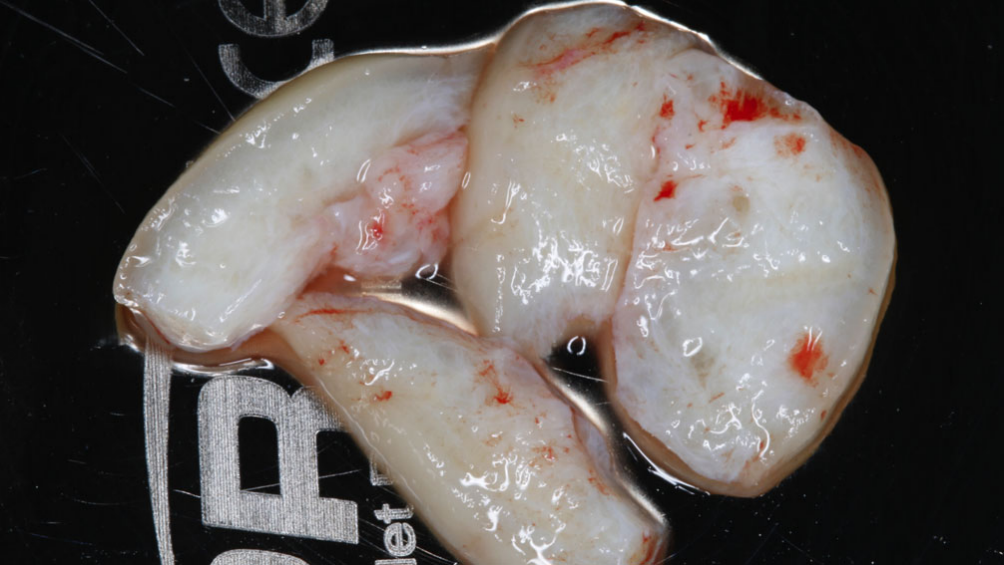
Connective tissue graft from tuberosity

**FIGURE 4:**
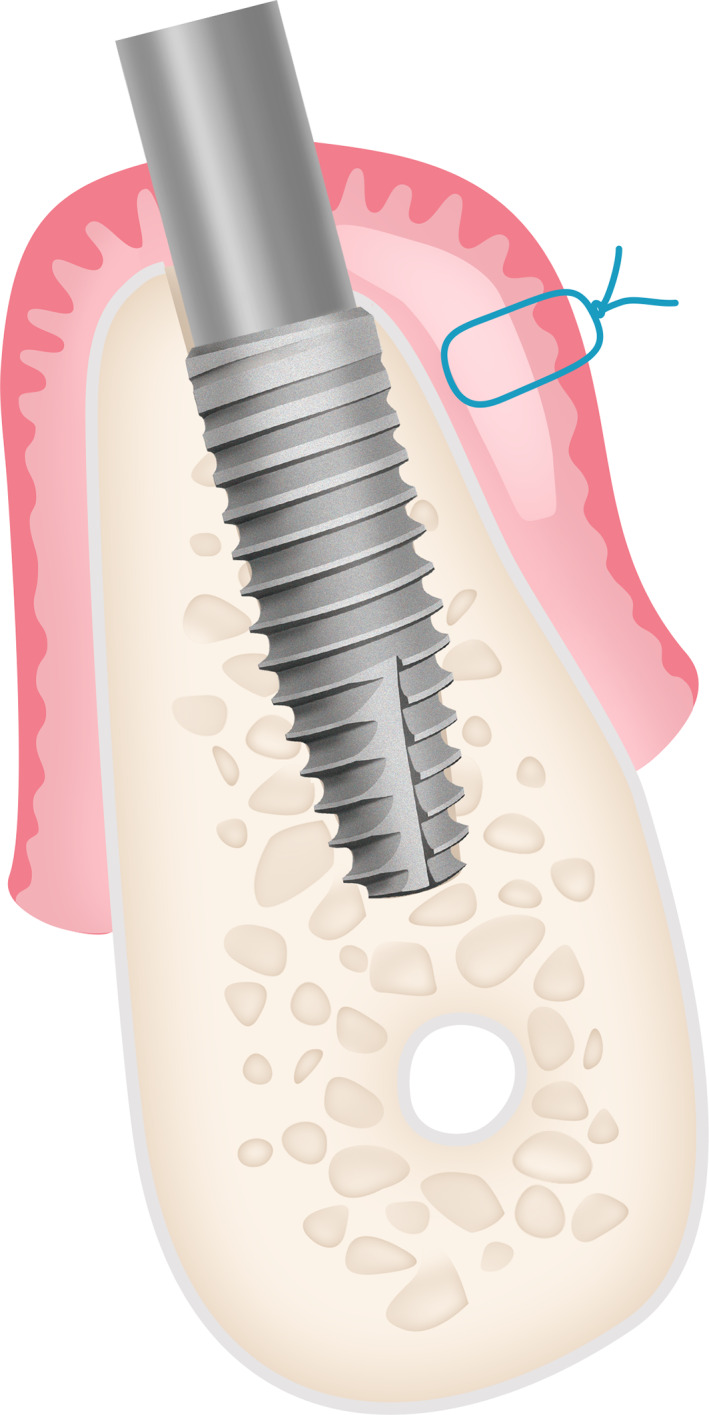
Illustration of partial thickness flap group

**FIGURE 5:**
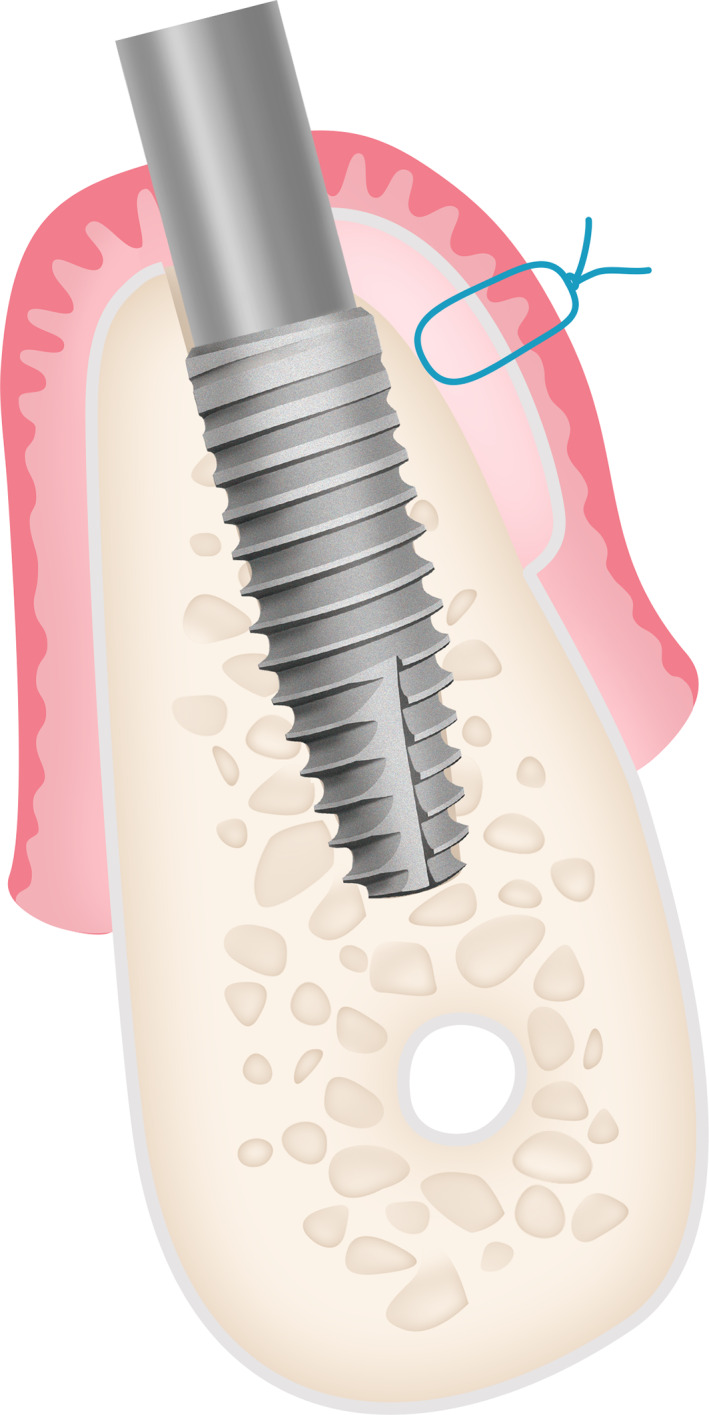
Illustration of full thickness flap group

**FIGURE 6:**
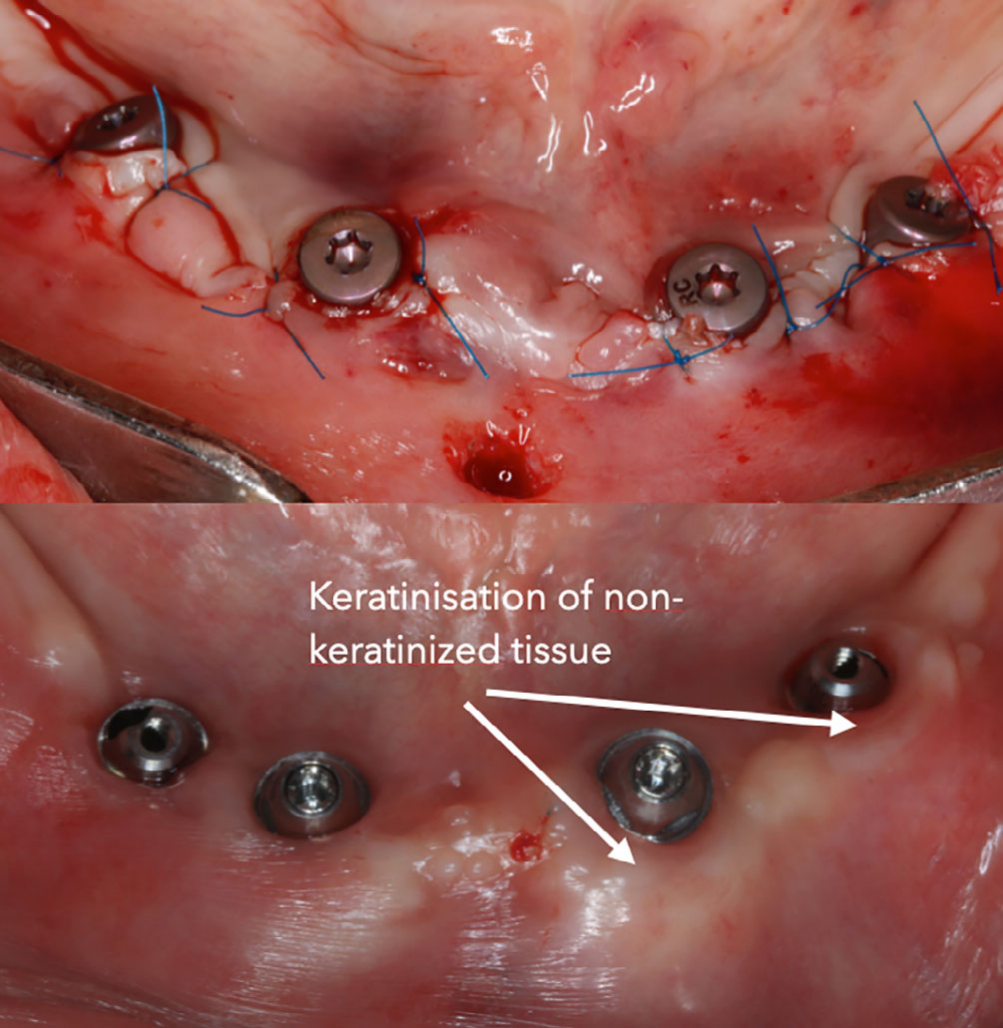
Placement of CTG and keratinization of non‐keratinized mucosa after 3‐year

Patients received a prophylactic dose of 2.0 g amoxicillin (Ospamox; Biochemie, Kiel, Germany) 1 h before the surgery. After the administration of 4.0% articaine, 40 mL solution (Ubistesin; 3 M ESPE, Seefeld, Germany) as local anesthesia, a surgical guide was fixed using bite registration. In the planned flapless procedure the soft tissues were perforated using a tissue punch and implant beds prepared following manufacturer's recommendations. Four bone level implants 4.1 mm diameter with the length of 12 or 14 mm (Bone Level Tapered, Roxolid SLActive, Straumann, Switzeland) were placed between the mental foramina in each patient: two implants mesially in straight position and two implants distally being tilted 30°. The implants were all positioned 4.0 mm deeper to the gingiva margin. Implant depth was controlled by standardized and marked implant insertion adaptor (Loxim®, Straumann, Switzerland). A primary stability of at least 35 N/cm was achieved in all cases. Multi‐unit abutments (Screw‐Ret.Abut.,TAN, Straumann, Switzeland) measuring 4.0 mm in height were attached and tightened at 35 N/cm. Four CTG were harvested bilaterally from the tuberosities after local anesthesia. The grafts dimensions were as follows: 2.0–3.0 mm thickness, 5.0–7.0 mm width and 8.0–10 mm length. In accordance with the randomization schedule either partial thickness (N 20) or full thickness (N 20) tunnel was prepared on the buccal site of each implant and the graft was positioned either on or sub periosteum and secured with 6–0 non‐resorbable sutures (Prolene, Jonson&Jonson) as it may be seen in Figures 2, 3, 4, 5, 6. Micro blade (MIM64, Hu‐friedy, USA) was used to make split thickness flap and micro papilla elevator (PH26M, Hu‐friedy, USA) was used to create a tunnel under periosteum. All surgeries were performed by one surgeon A.P.

Impressions and bite registrations were taken immediately after the operation and a relined fixed temporary prosthesis prepared in the prosthetic lab was delivered on the same day.

Postoperative medication included analgetics (Ibuprofen 400 mg 3x/day, 5 days US Pharmacia Sp.zo.o), 0.12% chlorhexidine digluconate (Perio‐aid; Dentaid, Spain) mouth rinse (2×/day for 7 days), and prophylactic antibiotics dose (Amoxicillin 1000 mg, 3 days, twice a day). Sutures were removed after 7 days, followed by a thorough cleaning of the prosthesis in the dental lab. Hygiene maintenance instructions were also given to the patients. A silicon impression was taken 2 months postoperatively, using an individual tray with an open‐tray technique. Final screw‐retained acrylic‐based prosthesis with metal frame restorations were delivered. In addition, periapical X‐rays were made using a Rinn‐like film holder after implant placement, 2 months after healing and at 1 year and 3 years follow up.

#### 2.3.3 Measurements

The primary outcome parameter of this randomized clinical trial was to evaluate the width increase of keratinized tissue (KT) and consequently to assess the keratinization (differentiation) potential of non‐keratinized gingiva. KT width were measured using periodontal probe with 1.0 mm calibration (UNC‐15 periodontal probe (Hu‐Friedy) applying functional method: the mobility of the tissues were determined by placing a horizontally positioned periodontal probe from the vestibule near the gingival margin using gentle pressure.

The secondary outcome parameters were to rate the changes of soft tissue thickness, the depth of recession, marginal bone level and general implant success. Measurements were taken immediately after the operation (baseline), after 2 months of healing, after 1 year and after 3 years.

The soft tissue thickness was measured by the help of an endodontic file and this measurement was performed 5.0 mm below the multi‐unit abutment margin at buccal site. A standardized caliper was used (Absolute Snap Caliper Series 573, Mitutoyo) and measurements were recorded to the nearest 0.1 mm mark.

The margin at multi‐unit buccal aspect was taken as a reference point to calculate midfacial soft tissue level. In the tabulation system used a recession appeared in a positive range and a gain of soft tissue in a negative one.

Periapical X‐rays were taken using a Rinn‐like film holder immediately after implant placement, 2 months after healing, and at 1 and 3 year follow‐up.

A surviving implant was defined as an implant in place at the time of the follow‐up. A particular implant was considered successful if all of the following success criteria (according to Buser et al. 1991) applied:

- The absence of persisting subjective discomfort such as pain, foreign body perception and/or dysesthesia (painful sensation).
- The absence of a recurrent peri‐implant infection with suppuration (where an infection is termed recurrent if observed at two or more follow‐up visits after the treatment with systemic antibiotics).
- The absence of implant mobility on manual palpation.
- The absence of any continuous peri‐implant radiolucency.

### 2.4 Statistical analysis

*One*‐*way ANOVA* with *repeated measures* in SPSS Statistics was used to evaluate mean differences in full and partial thickness groups at four time‐points (at baseline, after 2 months, 1 year and 3 years). A two‐tailed p‐value less than 0.05 was considered to be significant. The statistical analysis was performed using R statistical software.

## 3 RESULTS

Fourty implants were placed in 10 edentulous patients (2 males and 8 females, 65 (7,5) year old) presenting with atrophied mandibles (Class IV) and less than 1.0 mm of keratinized tissue. A flapless approach was preferred allowing an immediate rehabilitation with temporary acrylic bridge on multi‐unit abutments. All implants were 4.1 mm diameter. Twenty‐eight of 40 (70%) were 12 mm length, 12 (30%) of 40–14 mm length.

No complications of donor and recipient sites were observed, primary wound healing was uneventful. No other adverse events were observed during follow up time. No implant was lost. As all implants were in place after 3 years, fulfilling the success criteria of Buser (Buser et al., 1990), the implant survival rate was 100%.

Furthermore no prosthetic problems were identified at follow‐up visits.

After a 3 year follow‐up period the increase of KT was significantly higher in the partial thickness group, from 0.6(0.6) to 5.1(0.72) mm, comparatively to the full thickness group which showed very little growth from 0.5(0.51) to 1.0(0.57) mm. The difference between the groups was statistically significant (*p* < 0,001) at the end point of time.

STT was significantly increased in both groups over time: from 2.4(0.88) to 5.4(0.68) mm in full thickness group and from 2.5(0.51) to 5.8(0.41) mm in partial thickness group without any statistical significant difference between the groups.

Vertical recession was 2.6(0.7) mm in the full thickness group and 1.4(0.6) mm in the partial thickness one.

STT, the width of KT and vertical recession changes from baseline to the 3 year follow‐up are shown in Table 1.

**TABLE 1:** Repeated measures ANOVA model for STT, KT, and vertical recession parameters in full and Split thickness groups

Parameter	Timepoint	Full thickness mean (SD) median (min‐max)	Split thickness mean (SD) median (min‐max)	*P*‐value for time effect	*P*‐value for time*group effect
STT	Baseline	2.4 (0.88)	2.5 (0.51)	<0.001	0.228
		2 (1–4)	2 (2–3)		
	2 months	4.2 (0.83)	4.6 (0.56)		
		4 (3–6)	5 (3.5–5.5)		
	1 year	5.3 (0.66)	5.6 (0.5)		
		5 (4–6)	6 (5–6)		
	2 years	5.4 (0.68)	5.8 (0.41)		
		5.5 (4–6)	6 (5–6)		
KT	Baseline	0.5 (0.51)	0.6 (0.6)	<0.001	<0.001
		0 (0–1)	0.5 (0–2)		
	2 months	0.8 (0.7)	0.7 (0.57)		
		1 (0–2)	1 (0–2)		
	1 year	1 (0.58)	4.6 (0.94)		
		1 (0–2)	4 (3–7)		
	2 years	1 (0.57)	5.1 (0.72)		
		1 (0–2)	5 (4–7)		
Vertical recession	Baseline	0.4 (0.81)	−0.2 (0.62)	<0.001	0.001
		0 (1–2)	0 (−1 −1)		
	2 months	1.5 (1.01)	0.4 (0.59)		
		1 (0–4)	0 (−1 −1)		
	1 year	2.2 (0.71)	1.1 (0.45)		
		2 (1–4)	1.2 1 (0–2)		
	2 years	2.6 (0.7)	1.4 (0.6)		
		2.75 (1.5–4)	1 (0–2)		

Because of the totally atrophied alveolar bone it was not possible to achieve periapical x‐rays with a sufficient reproducibility, thus, a precise visualization of the crestal bone was simply not possible. The bone levels were measured on panoramic X‐ray once a 3 year follow‐up period. Eight out of 40 implants showed more than 1.0 mm bone loss, but not more than 2.0 mm without any specific occurrence between the groups or at distal tilted implants. No difference between the straight and tilted implants were noticed.

## 4 DISCUSSION

It has to be outlined that if the number of patients is adequate for a pilot study and although the principal outcome showed with statistical significance, larger trials including more patients are needed. It is clear, no strict clinical recommendations could be offered from only 10 patients. Another limitation of this pilot study is the fact that due to the atrophy of alveolar ridge it was not possible to get parallel X‐rays using the Rinn like holder. Consequently, a suitable evaluation of the crestal bone level was not possible. Taking into consideration the above conditions this study remains unique as it demonstrates clearly, that CTG harvested from the tuberosity has the potential to keratinize and determine the epithelial differentiation of non‐keratinized tissues when placed just underneath a thin mucosa and above the periosteum. Thus, our initial null hypothesis is validated. According to the authors best knowledge, this is the first study having evaluated the differences between two CTG insertion and stabilization methods under oral soft tissues.

This process of keratinization was described first by Karring et al. (1975). Free grafts without epithelium were transferred from either the keratinized gingiva or the non‐keratinized mucosa into areas of alveolar mucosa. After 1 month, clinical and histological findings showed that the gingival CTG became covered with keratinized epithelium, indicating that CTGs are able to determine the differentiation of non‐keratinized mucosa to keratinized tissue.

The explanation of this tendency to keratinize may be found in the structure and biological differences of CTG. Macroscopic and microscopic investigations showed, that CTGs from the tuberosity present with less blood vessels, less fatty glandular tissue, higher density of collagen fibers and more maturated fibroblasts comparatively to CTG from palate (Studer et al., 1997). Hence, a genetic determination of tendency for keratinization was previously reported (Dellavia et al., 2014).

In fact, a superficial layer of de‐epithelized gingival graft form palate seems to have similar features to a graft obtained from the tuberosity. Furthermore, it was also concluded, what the variety between studied individuals was very large (Bertl et al., 2015).

At this stage we have to question the trigger elements and the process of keratinization of oral soft tissues. Taking into consideration genetic potentials, variations between individuals and donor sites and surgical placement techniques of different sizes of CTG into different sites under different soft tissue biotypes and thickness, evaluation methods, all renders an explanation difficult and even controversial. This could explain, why keratinization occurs or is recorded variously depending on study design, surgical attention and evaluation methods.

In the present study it is interesting to notice that after 2 months of healing there was no difference between the two groups, but a significant change was clearly identified after 1 year. This corroborates the idea that the time is a decisive factor for the differentiation of the mucosa structure.

In this study the keratinization element correlates with the elevation and suturing of a partial thickness flap. In other words, where dense CTG was covered with thin superficial non‐keratinized mucosa. According to soft tissue thickness changes, the gain was similar within both groups, indicating a possible hyperplastic response. This is in agreement with other authors who observed similar results. Jung et al found that CTG from tuberosity progressively tended to a hyperplastic appearance and led to non‐aesthetic soft tissue changes after root recession coverage procedures (Jung et al., 2008). Resolving and improvement of these non‐aesthetic results may be demanding, according to some authors (Gluckman et al., 2019). Bertl et al. concluded, that it is advisable to thin the CTG and calculate a graft growth around 30–40% (Bertl et al., 2015).

A protective clinical mindset suggests to place the CTG as close as possible to the bone, leaving as much as possible thicker flap over the graft if only soft tissue thickening is needed. And vice versa, to place it under a partial thickness flap as thin as possible if KT is needed. It remains unclear, how thick the covering soft tissue flap should be. This was the reason to compare partial thickness and full thickness flaps. If the periosteum covers the CTG, only a hyperplastic reaction without differentiation was noticed during the follow‐up examination periods. The possibility to place grafts on denuded bone was clinically shown in a few studies. Despite that, a majority of clinicians use only partial thickness approach (Puisys et al., 2015; Puišys et al., 2017; Puisys & Linkevicius, 2015; Wiesner et al., 2010).

## 5 CONCLUSION

With the limitations of this pilot study, it can still be concluded, that not only the graft properties lead to different potential of differentiation of epithelial cells, but also the applied placement method (full or partial thickness flap). Placing CTG from tuberosity while full thickness flap was prepared, leads to soft tissue thickening, placing CTG underneath a partial thickness flap, induces the occurrence of soft tissue thickening and keratinization of non‐keratinized mucosa.

### 5.1 Clinical implication and future research

The use of full thickness flaps in aesthetic area to avoid keratinization and changes of color and/or texture of soft tissue.

The use of partial thickness flap to enhance keratinization in areas where KT is needed, where both texture and color are less critical (lower jaw).

## CONFLICT OF INTEREST

Authors state no conflict of interest.

## AUTHOR CONTRIBUTIONS

Algirdas Puisys conceived the idea and placed the implants and followed the patients; Egle Vindasiute‐Narbute restored the implants; Saulius Zukauskas and Viktorija Auzbikaviciute collected the data; Dainius Razukevicus and Algirdas Puisys analyzed the data, drafted the paper; Michel M. Dard led the writing.

## Data Availability

The data that support the findings of this study are openly available in [repository name e.g “figshare”] at http://doi.org/[doi], reference number [reference number].
